# Exploring the Therapeutic Mechanism of Xiehuo Pingtu San in Treating Thyroid Eye Disease Based on Network Pharmacology, Molecular Docking, and Molecular Dynamics Simulation

**DOI:** 10.1155/ije/2727402

**Published:** 2026-02-12

**Authors:** Ping Wang, Ruiyan Liu, Xin Shang, Yu Fu, Ying Wang, Shuxun Yan

**Affiliations:** ^1^ Endocrinology Department, The First Affiliated Hospital of Henan University of CM, Zhengzhou, Henan, China; ^2^ School of Traditional Chinese Medicine, Beijing University of Traditional Chinese Medicine, Beijing, China, bucm.edu.cn; ^3^ Geriatric Cardiology Department, The First Affiliated Hospital of Zhengzhou University, Zhengzhou, Henan, China, zzu.edu.cn

**Keywords:** molecular docking, molecular dynamics simulation, network pharmacology, thyroid eye disease, Xiehuo Pingtu San

## Abstract

**Background:**

Xiehuo Pingtu San (XHPTS) has been shown to be safe and effective in treating thyroid eye disease (TED), yet its underlying mechanisms remain unclear. This study aimed to elucidate the active ingredients of XHPTS and their therapeutic mechanisms in TED through network pharmacology, molecular docking, and molecular dynamics simulations.

**Methods:**

Active ingredient targets for XHPTS were screened through the TCMSP and BATMAN‐TCM databases. TED‐related targets were obtained from GeneCards, OMIM, and CTD, and differentially expressed genes (DEGs) between TED patients and healthy controls were retrieved from GEO. The intersecting targets among active ingredient targets, disease targets, and DEGs were defined as key targets. Gene Ontology (GO) terms and Kyoto Encyclopedia of Genes and Genomes (KEGG) enrichment analyses were performed to identify biological processes and pathways associated with XHPTS intervention in TED. Key targets were further mapped to organs to predict potential “target–organ” interactions. A protein–protein interaction (PPI) network was used to identify hub genes. Immune microenvironment analysis was conducted to compare immune cell infiltration between TED and control samples and to assess correlations between differential immune cells and hub genes. A ceRNA network (“mRNA–miRNA–lncRNA”) was constructed based on hub genes. Molecular docking and molecular dynamics simulations were applied to evaluate the binding affinities between key active ingredients and hub genes.

**Results:**

We found that XHPTS may exert therapeutic effects on TED through biological functions, such as reactive oxygen species (ROS) responses and fatty acid (FA) metabolism, as well as signaling pathways, such as IL‐17. Several shared targets, such as ADIPOQ, CES1, and CAT, were identified across these pathways. Organ localization analysis indicated that the liver plays a crucial role in the therapeutic action of XHPTS against TED. Immune microenvironment analysis revealed significant differences in immune cell infiltration between TED patients and healthy individuals, particularly in plasma cells, and these differential immune cells were correlated with the identified hub genes. A ceRNA regulatory network revealed that 153 lncRNAs may regulate 8 miRNAs and 4 hub genes. Molecular docking and molecular dynamics simulations showed strong binding affinities between key active ingredients (quercetin, luteolin, and paeoniflorin) and hub genes (IL‐6, PPARγ, CXCL8, CAT, and CAV1). The binding free energies of key complexes ranged from −51.923 to −98.221 kJ/mol, confirming stable interactions.

**Conclusion:**

XHPTS exerts therapeutic effects on TED through multicomponent, multitarget, and multipathway approaches.

## 1. Introduction

Thyroid eye disease (TED) is a rare autoimmune orbital disease and the most common extrathyroid manifestation of Graves’ disease (GD) [1]. The current incidence rate of TED is approximately 0.54–0.90 cases per 100,000 people per year for men and 2.67–3.3 cases per 100,000 people per year for women [2]. Although the precise pathogenesis of TED remains unclear, immune cell involvement, oxidative stress (OS), gut microbiome dysregulation, and epigenetic abnormalities are recognized as key contributors to its development [1]. Histopathologically, TED is characterized by lymphocyte infiltration in orbital tissues, fibrosis of extraocular muscles (EOMs), and proliferation or remodeling of orbital adipose tissue. Clinically, TED presents with symptoms, such as proptosis, eyelid edema and/or retraction, EOM impairment, orbital deformities, diplopia, and in severe cases, blindness [3]. TED significantly affects patients’ quality of life, mental health, and financial well‐being [4]. Current treatments for TED include steroids, immunosuppressants, statins, biologics, and surgical interventions. However, challenges remain, including limited efficacy, numerous side effects, high recurrence rates, and the high cost of medications [5, 6]. Therefore, elucidating the underlying mechanisms of TED and developing novel therapeutic approaches are critical for improving clinical management.

Traditional Chinese Medicine (TCM) has a long history of clinical application, offering multitarget, multipathway, and multistage therapeutic advantages. In TCM theory, TED corresponds to conditions described as “red gaze resembling a hawk” or “protruding eyes,” and is believed to arise from dysfunctions of the liver, spleen, and kidney [7]. TCM therapy has shown benefits in reducing complications, mitigating the side effects of Western medications, shortening treatment duration, and providing comprehensive systemic regulation [8]. Xiehuo Pingtu San (XHPTS), a classical TCM formula used for TED with liver fire syndrome, has been applied for many years in the Endocrinology Department of the First Affiliated Hospital of Henan University of Chinese Medicine and has received a national invention patent (CN201310402152.X). Clinical studies have demonstrated that XHPTS can reduce TCM symptom scores, Clinical Activity Scores (CAS), and NOSPECS grading in TED patients [9]. Additionally, XHPTS‐containing serum has been shown to inhibit the proliferation of TED orbital fibroblasts (OFs) and suppress the overexpression of intercellular adhesion molecule‐1 (ICAM‐1), human leukocyte antigen‐DR (HLA‐DR), and CD40. It also reduces the synthesis of hyaluronic acid (HA) and collagen types I and III [10], while restoring the balance between matrix metalloproteinases (MMPs) and tissue inhibitors of metalloproteinases (TIMPs) [11]. However, the molecular mechanisms through which XHPTS exerts therapeutic effects on TED remain unclear.

NP provides an important link between TCM and modern pharmacology by constructing a “drug–target–disease” network. This framework helps clarify how biological systems, therapeutic compounds, and diseases interact from a systems‐level perspective, which is consistent with the holistic principles of TCM [12]. By identifying active components, characterizing target interactions, and constructing molecular regulatory networks, NP enables a deeper understanding of the pharmacological mechanisms underlying TCM therapies [13].

**FIGURE 1:**
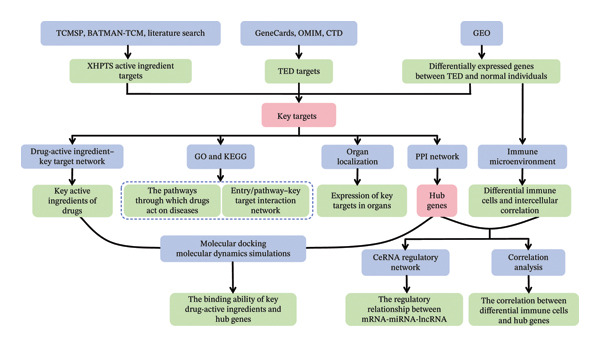
Overall workflow of the study.

In summary, this study revealed the therapeutic mechanism of XHPTS in treating TED through NP and validated it through molecular docking and dynamic simulation analysis. These results provide a foundation for future experimental and clinical investigations. The overall workflow is shown in Figure 1. The main contributions of this study are as follows:

1. The TCMSP, BATMAN‐TCM, and UniProt databases, together with a literature review, were used to identify the active ingredients of XHPTS and their corresponding targets. TED‐related disease targets were obtained from GeneCards, OMIM, and CTD, and differentially expressed genes (DEGs) between TED and normal samples were identified using the GEO database.
2. Key targets and key active ingredients were identified by intersecting active ingredient targets, disease targets, and DEGs.
3. Enrichment analyses were performed to evaluate the distribution of key targets in Gene Ontology (GO) terms and Kyoto Encyclopedia of Genes and Genomes (KEGG) pathways. Shared targets were identified by constructing an “item or pathway–key target” network.
4. Organ localization analysis was conducted to determine the organ distribution of key targets and to predict highly expressed genes within the corresponding target–organ associations.
5. A protein–protein interaction (PPI) network was constructed to identify hub genes involved in the interaction between XHPTS and TED.
6. Immune microenvironment analysis based on the hub genes was performed to identify differential immune cell populations between TED and normal samples, and Pearson correlation analysis was used to investigate the relationships between these immune cells and the hub genes.
7. The miRWalk, miRDB, Starbase, and miRNet databases were used to construct a competing endogenous RNA (ceRNA) regulatory network. Shared miRNAs and lncRNAs related to the hub genes were identified, and the regulatory relationships within the “mRNA–miRNA–lncRNA” network were established.
8. Molecular docking and molecular dynamics simulations were conducted to assess the interactions between the key active ingredients of XHPTS and the identified hub genes.

## 2. Materials and Methods

### 2.1. XHPTS Active Ingredient and Target Screening

The active ingredients of XHPTS and their corresponding targets were identified using the TCM Systems Pharmacology Database and Analysis Platform (TCMSP) (V2.3, http://lsp.nwu.edu.cn/tcmsp.php) [14] and the BATMAN‐TCM (2021 update, V2.0, http://bionet.ncpsb.org/batman-tcm/) online analysis tool library [15]. The herbs included in the XHPTS formula are Gentiana Radix Et Rhizoma (Long Dan Cao, LDC), Gardeniae Fructus (Zhizi, ZZ), Scutellariae Radix (Huangqin, HQ), Prunellae Spica (Xiakucao, XKC), Chrysanthemi Flos (Juhua, JH), Celosia Semen (Qing Xiang Zi, QXZ), Dioscorea Bulbifera (Huang Yao Zi, HYZ), Cortex Moutan (Mu Dan Pi, MDP), Radix Paeoniae Rubra (Chishao, CS), Indigo Naturalis (Qing Dai, QD), and Tribuli Fructus (Baijili, BJL). In the TCMSP database, the search terms were the Latin names of the above herbs. Active ingredients were screened based on ADME (absorption, distribution, metabolism, and excretion) parameters. The criteria for selection were set as follows: oral bioavailability (OB) ≥ 30% and druglikeness (DL) ≥ 0.18. The BATMAN‐TCM database was queried using the same herb names. The inclusion parameters were set as a score cutoff of at least 20 for drug–target interactions and an adjusted *p*‐value threshold of 0.05. Unforeseen active ingredients and their targets were supplemented through a literature review of published research. The UniProt protein database (2023.03 update, https://www.uniprot.org) [16] was used to standardize the protein target information. The inclusion criteria required that targets belong to the species Homo sapiens and be annotated as protein‐coding. Exclusion criteria included nonhuman homologous proteins and targets lacking functional annotation.

### 2.2. TED Target Prediction

The disease‐related targets of TED were identified using the search terms “Thyroid eye disease (TED)” and “Thyroid‐associated ophthalmopathy (TAO)” in the GeneCards database (2023.1 update, http://www.genecards.org/) [17], with the species constraint set to Homo sapiens. The search was supplemented with data from the Online Mendelian Inheritance in Man (OMIM) database (2023.03 update, http://omim.org/) [18] and the Comparative Toxicogenomics Database (CTD) (2023.04 update, http://ctdbase.org/) [19]. In the GeneCards database, the Category parameter was restricted to protein coding. Disease relevance was evaluated based on the score values provided by the database, with higher scores indicating stronger associations with TED. Only those with scores above the median value (GeneCards median score = 12.3) were selected as potential TED targets due to the excessive number of initial targets identified. The GeneCards, OMIM, and CTD targets were merged, and duplicate entries were removed to create a unified set of TED‐related targets.

### 2.3. Identification of DEGs

The GEO database (https://www.ncbi.nlm.nih.gov/geo/) [20] was searched using the terms “TED,” “TAO,” and “Homo sapiens” (species filter). The GSE58331 dataset was selected for analysis, and matrix and platform data files were downloaded. Differential gene expression between TED patient samples and healthy controls was analyzed using the R package limma (V 3.42.2). Both upregulated and downregulated DEGs were identified based on the criteria |log_2_ fold change| > 0.5 and *p* < 0.05. A heatmap of the DEGs was generated using the pheatmap package (V 1.0.12).

### 2.4. Construction of Drug‐Active Ingredient–Key Target Network

To identify potential key targets for XHPTS treatment of TED, the active ingredient targets of XHPTS were intersected with TED disease targets and DEGs. A Venn diagram was generated using R 4.0.3 software to visualize the overlap between these sets. The drug, active ingredients, and key targets were imported into Cytoscape 3.6.1 to create a “drug‐active ingredient‐key target” network diagram. Free nodes were removed, and network topology analysis was performed utilizing the Network Analyzer plugin to identify the key active ingredients of XHPTS for treating TED.

### 2.5. GO Function and KEGG Pathway Enrichment Analysis, and Construction of Item/Pathway–Key Target Network

The GO system is composed of three categories: Biological Process (BP), Molecular Function (MF), and Cellular Component (CC) [21]. The KEGG integrates functional information across genomes, proteomes, and chemical compositions [22]. GO and KEGG enrichment analyses were performed using the Database for Annotation, Visualization and Integrated Discovery (DAVID) (V6.8, https://david.ncifcrf.gov/) [23] and the R language package “clusterProfiler” to analyze the possible biological functions and pathways involved in key targets. The identifier was set to “official gene symbol,” the list type to “gene list,” and the species to “Homo sapiens.” Items and pathways with *p* < 0.05 were considered significantly enriched. The enrichment results were visualized using the R package ggplot2. Genes and their corresponding entries from the top‐ranked GO and KEGG results were extracted and imported into Cytoscape 3.6.1 to construct the item or pathway–key target network.

### 2.6. Organ Localization Analysis

Organ localization analysis for key targets was conducted using the eFP browser database (V8 http://bar.utoronto.ca/efp_human/cgi-bin/efpWeb.cgi). Normalized expression values (TPM) of 18 key targets across 54 human tissues were extracted, and tissue specificity score (Tau) was calculated via the R package “pheatmap” (v1.0.12). Relevant organs were defined as those with average TPM > 5 and Tau > 0.3.

### 2.7. Construction of PPI Network and Hub Gene Screening

PPI analysis of the key targets was performed using the STRING platform (v11.5, https://string-db.org) [24]. The species was set as “Homo sapiens” with a confidence score of ≥ 0.4, and discrete proteins were removed to generate the PPI network related to XHPTS treatment for TED. The results were saved in “tsv” format and imported into Cytoscape 3.6.1 to create a PPI network diagram. Three complementary network‐based algorithms were employed for core target screening: Degree Centrality, CytoHubba (Maximal Clique Centrality, MCC), and MODEX [25–27]. The top five genes ranked by degree were selected as hub genes.

### 2.8. Immune Microenvironment Analysis

The CIBERSORT algorithm combined with the LM22 gene set was used to estimate the proportions of immune cell types in TED and normal samples. Samples with *p* > 0.05 were excluded. A heatmap was generated to visualize immune cell infiltration patterns across all samples. The R package vioplot was used to produce a violin plot based on the Wilcoxon test to identify immune cells that differed significantly between TED and normal samples. Pearson’s correlation analysis was performed on the expression levels of these immune cells using the R package “corrplot,” and the correlation between immune cells and hub genes was calculated using the Spearman method.

### 2.9. Analysis of ceRNA Regulatory Network

miRNA targeting of hub genes was predicted using the miRWalk (V3.0 http://mirwalk.umm.uni-heidelberg.de/) [28] and miRDB (V6.0 https://mirdb.org/mirdb/index.html) [29] databases. The parameters were set as follows: score = 0.95, position = 3UTR, and binding probability ≥ 1 in miRWalk; target score ≥ 80 in miRDB. The predicted miRNAs from both databases were intersected to identify shared miRNAs. Starbase (V3.0 http://starbase.sysu.edu.cn/) [30] and miRNet (https://www.mirnet.ca/miRNet/home.xhtml) [31] were utilized to predict lncRNAs interacting with the shared miRNAs, and the intersections of the predicted lncRNAs were used to obtain shared lncRNAs. Regulatory relationships between miRNAs and hub genes were extracted based on shared lncRNAs, and the mRNA‐miRNA‐lncRNA network was visualized using Cytoscape.

### 2.10. Molecular Docking

The purpose of molecular docking was to predict the binding mode and affinity between key active ingredients of XHPTS and hub genes associated with TED to verify their potential direct interaction. For ligand preparation, the 3D structures of key active ingredients were retrieved from the PubChem database (https://pubchem.ncbi.nlm.nih.gov/) [32], preprocessed using Open Babel 3.1.1 (hydrogen atom addition, bond order correction, energy minimization via conjugate gradient method with 1000 iterations, Gasteiger charge assignment, and rotatable bond definition), and converted to.pdbqt format. The crystal structures of hub genes were downloaded from the RCSB Protein Data Bank (https://www.rcsb.org/) [33], processed with AutoDockTools 1.5.6 (water molecule and endogenous ligand removal, polar hydrogen atom addition, and Kollman charge assignment), and saved as.pdbqt format after structural optimization. Molecular docking was performed using AutoDock Vina 1.2.0, with the docking box centered on each receptor’s active pocket (grid size: 30 × 30 × 30 A, spacing = 1 A) and exhaustiveness set to 100, and a binding energy threshold of ≤ −7 kcal/mol was used to judge high‐affinity interactions. To clarify specific binding relationships, targeted docking pairs were set, with each ligand docked only to its corresponding target protein. Select two clinically validated drugs as positive controls (such as tocilizumab and pioglitazone) and DMSO as negative control to verify the reliability of docking. Docking results were evaluated based on binding energy and interaction mode (complexes with binding energy ≤ −7 kcal/mol were considered stably bound), and top‐ranked conformations were visualized using PyMOL 2.5.2 to analyze key interactions (hydrogen bonds, hydrophobic interactions, and π‐π stacking).

### 2.11. Molecular Dynamics Simulation

The root mean square deviation (RMSD) was used to evaluate the coordinate deviations of selected atoms relative to the reference structure and to determine whether the simulation system had reached equilibrium. Solvent accessible surface area (SASA) was analyzed to assess changes in protein surface exposure during the simulation. The radius of gyration (Rg) was calculated to examine the compactness of the protein structure. The root mean square fluctuation (RMSF) was used to assess the flexibility of different protein regions by analyzing atomic fluctuations relative to their mean positions. Hydrogen bond analysis was performed to characterize the interactions between proteins and ligands. Molecular mechanics Poisson–Boltzmann surface area (MM‐PBSA) was employed to calculate the binding free energy between proteins and ligands after molecular dynamics simulation. Binding free energy consists of three components: molecular mechanics energy (MM), including van der Waals forces and Coulomb electrostatic interaction energy; polarization solvation energy (PB); and nonpolar solvation energy (SA). Force field: Amber14SB; water model: TIP3P; ion concentration: 0.15 M NaCl; and temperature: 300 K. The binding free energy reflects the stability of ligand–receptor interactions, and the gmx_MMPBSA 1.5.1 was used to calculate the binding free energy after equilibrium. Lower binding free energy indicates stronger receptor–ligand affinity. Decomposition of the total binding free energy by residue provided a detailed visualization of individual interaction contributions.

## 3. Results

### 3.1. Active Ingredients and Targets of XHPTS

A search of the TCMSP and BATMAN‐TCM databases and a literature review identified 187 chemical components in XHPTS. After removing duplicates and compounds lacking target information, 90 active ingredients were retained. Using the target normalization using the UniProt database resulted in 248 active ingredient–associated genes.

### 3.2. Target of TED

A total of 9,134, 200, and 1785 TED‐related targets were retrieved from the GeneCards, OMIM, and CTD databases, respectively. After merging these datasets and removing duplicate entries, 9758 unique TED‐related targets were obtained (Figure 2(a)).

FIGURE 2TED target, DEGs between diseases and normal samples, as well as the interaction between drug‐active ingredients and them. (a) TED target Venn diagram. (b) Volcano plot of DEGs between TED and normal samples. (Each point represents a gene, with red indicating upregulated genes, green indicating downregulated genes, and gray indicating genes with no significant changes.) (c) Heatmap of the top 50 DEGs between TED and normal samples. (Each square represents a gene, with darker colors indicating higher expression levels. Red represents high expression, while blue indicates low expression. The tree diagram on the left shows the clustering of genes across samples.) (d) Venn diagram of active ingredient targets, disease targets, and DEGs. (e) Drug‐active ingredient–key target interaction network diagram (green nodes represent 11 drugs, yellow nodes represent 23 active ingredients, and purple nodes represent 18 key targets).

**Figure:**
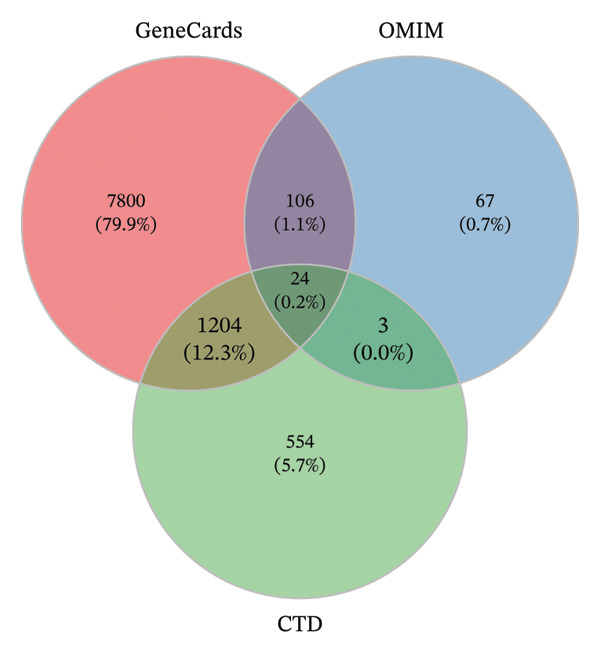
(a)

**Figure:**
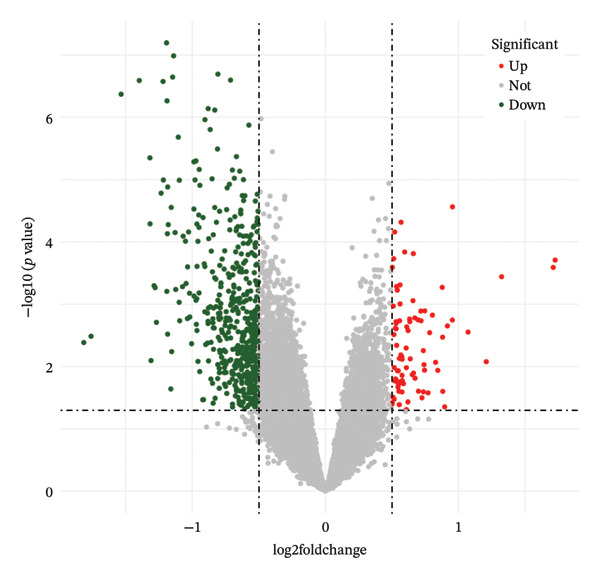
(b)

**Figure:**
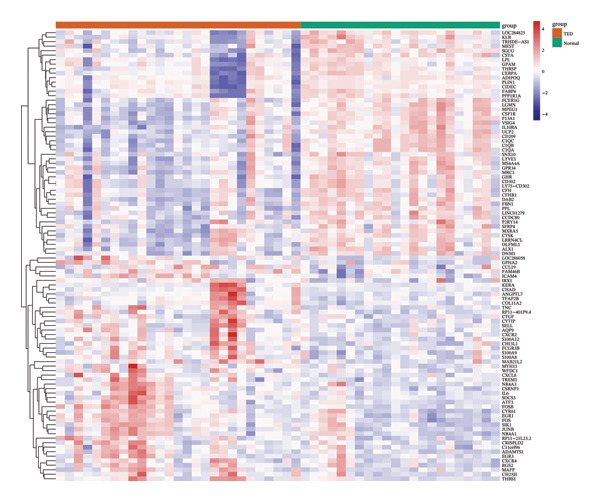
(c)

**Figure:**
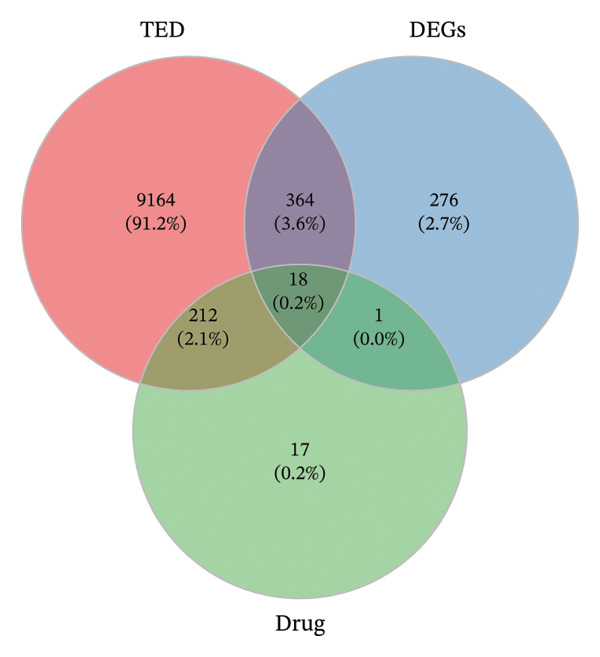
(d)

**Figure:**
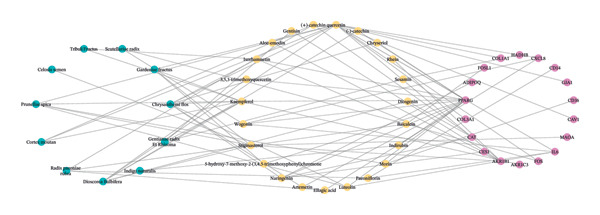
(e)

### 3.3. Identification of DEGs

A differential expression analysis of 27 TED patient samples and 22 normal samples from the GSE58331 dataset revealed 659 DEGs. Among these, 86 genes were upregulated (top‐ranked: *FOS, EGR1, CHAD, CYR61, CSRNP1*) and 573 were downregulated (top‐ranked: *PLIN1, ADIPOQ, LGMN, CIDEC, FABP4*). Volcano plots of the DEGs and heatmaps of the top 50 upregulated and downregulated genes were generated (Figures 2(b), 2(c)).

### 3.4. Construction of Drug‐Active Ingredient–Key Target Network

By intersecting the 248 active ingredient target genes, 9758 disease targets, and 659 DEGs, 18 intersection targets were identified as key targets (Figure 2(d)). A “Drug‐Active Ingredient‐Key Target” network for XHPTS in TED treatment was constructed using Cytoscape 3.6.1, which included 18 key targets and their corresponding 23 active ingredients (Figure 2(e)). The top‐ranked active ingredients were quercetin, luteolin, paeoniflorin, ellagic acid, naringenin, and (+)‐catechin, which were designated as the key active ingredients. These ingredients play a critical role in XHPTS’s therapeutic effect on TED.

### 3.5. GO Function and KEGG Pathway Enrichment Analysis, and Construction of Item/Pathway–Key Target Network

GO annotation and KEGG functional enrichment were performed on the 18 key targets, identifying 717 BP, 20 CC, 46 MF, and 29 KEGG signaling pathways. The top 10 results for GO and KEGG were visualized using the R package “ggplot2.” The genes were significantly enriched in BP categories, such as response to reactive oxygen species (ROS), fatty acid (FA) metabolic process, and response to alcohol; in CC categories including fiber collagen trimer, banded collagen fiber, and collagen trimer; and in MF categories, such as platelet‐derived growth factor (PDGF) binding, alditol+1‐oxidoreductase activity, and NADP‐retinol dehydrogenase activity(Figures 3(a), 3(b)). KEGG pathway enrichment highlighted pathways, such as lipid and atherosclerosis, nonalcoholic fatty liver disease (NAFLD), pertussis, and IL‐17 signaling (Figures 3(c), 3(d)).

FIGURE 3GO and KEGG analysis. a‐b: GO enrichment results. (The vertical axis represents enriched GO items, with different colors representing different *p* values, and colors from blue to red indicating increased significance. (a) Bar lengths represent the number of genes in each term. (b) Dot sizes represent the number of genes in each term.) c‐d: KEGG enrichment results (The vertical axis represents enriched KEGG projects, with longer bars or larger bubbles indicating more enriched targets in the pathway. The redder the bars or bubbles, the stronger the significance.) e‐f: Item/pathway–key gene interaction network (e: GO; the diagram consists of 47 points and 118 edges; orange circles represent key genes, while purple, red, and green circles represent Top10 BP, Top10 CC, and Top10 MF, respectively; f: KEGG; the diagram consists of 20 points and 42 edges; orange circles represent key genes, and red represents the top 10 KEGG signaling pathways).

**Figure:**
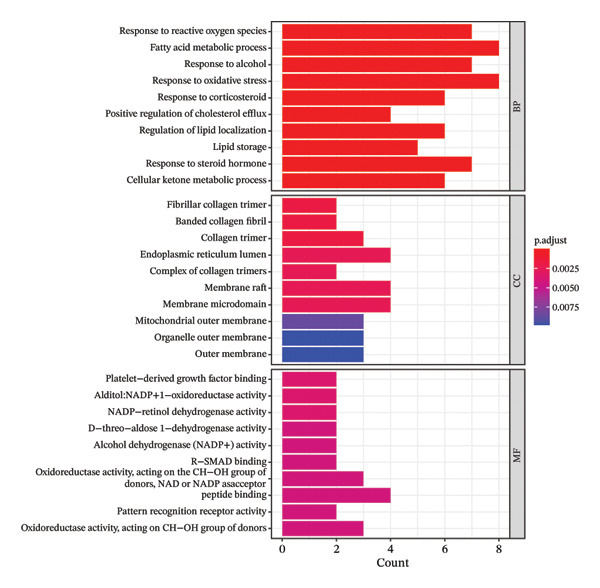
(a)

**Figure:**
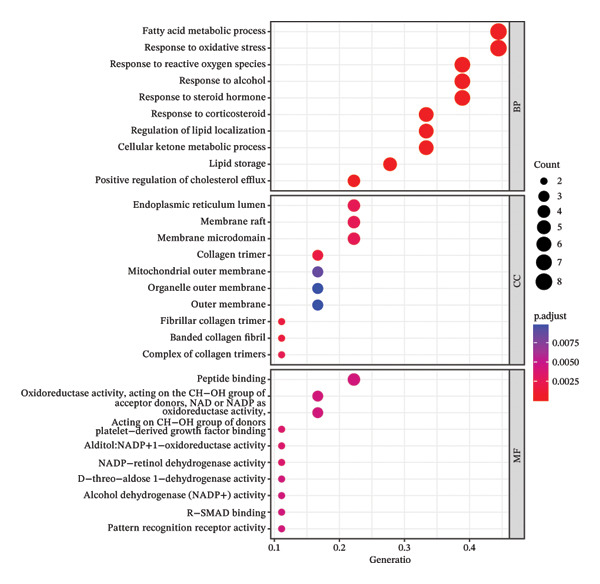
(b)

**Figure:**
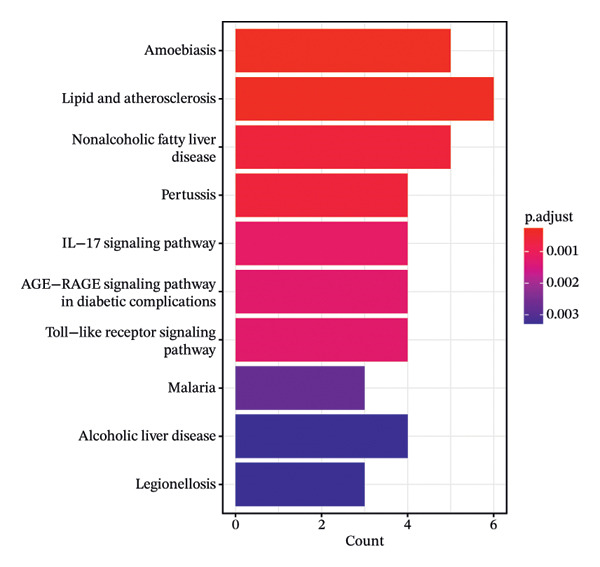
(c)

**Figure:**
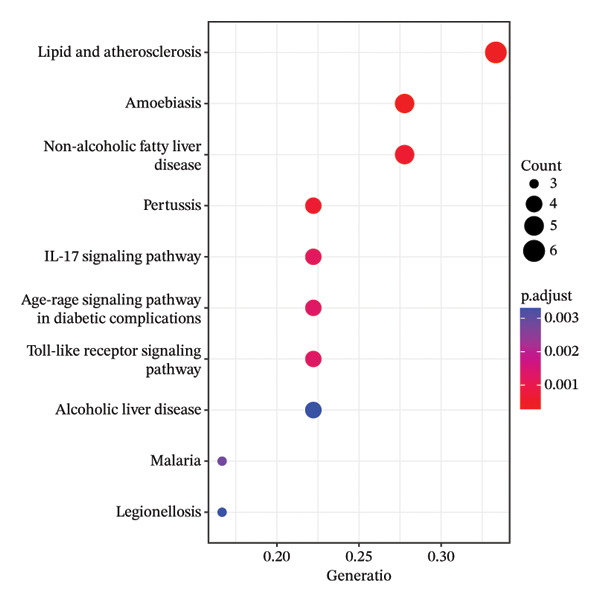
(d)

**Figure:**
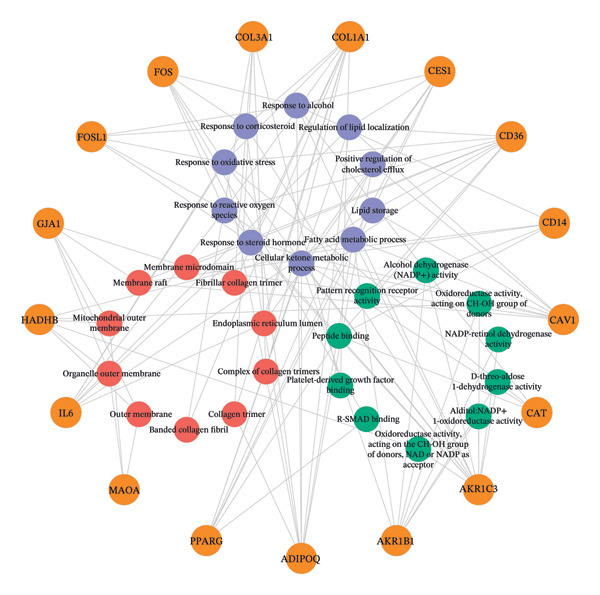
(e)

**Figure:**
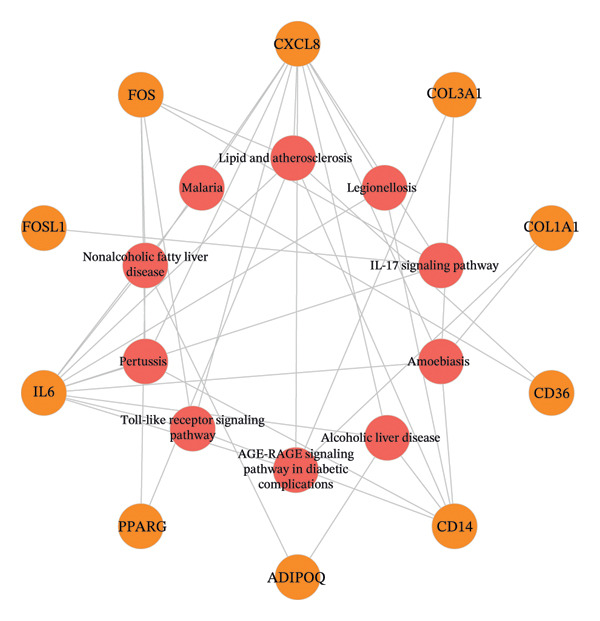
(f)

Genes and their corresponding entries from the top 30 GO terms and top 10 KEGG pathways were extracted to construct an item/pathway–key target network. Several shared targets were identified. ADIPOQ and CES1 were common to both the FA metabolic process and response to alcohol. CAT, FOS, and FOSL1 were shared among the responses to ROS and response to alcohol categories. COL1A1 and COL3A1 were shared across the fiber collagen trimer, the banded collagen fiber, and the collagen trimer. CD14 was shared by the lipid and atherosclerosis and pertussis pathways. CXCL8, IL‐6, and FOS appeared in lipid and atherosclerosis, NAFLD, pertussis, and IL‐17 signaling. PPARγ was shared by lipid and atherosclerosis and NAFLD (Figures 3(e), 3(f)).

### 3.6. Organ Localization Analysis of Key Targets

Organ localization analysis was conducted for the 18 key targets. In the liver, highly expressed genes included *IL-6*, *FOSL1*, and *CAV1*. In the heart, the highly expressed genes were *IL-6*, *FOSL1*, *CAV1*, *ADIPOQ*, and *HADHB*. *MAOA* showed high expression in the kidneys, *CES1* in the lungs, and *CAT*, *CD14*, *CXCL8*, and *FOS* in the blood (Figure 4(a)). To confirm these findings, the expression patterns of the 18 key targets were compared with data from the GTEx database. Consistent with the initial results, *IL-6*, *FOSL1*, and *CAV1* were significantly overexpressed in the liver (FDR < 0.01), supporting the central role of the liver in the therapeutic effects of XHPTS on TED.

FIGURE 4Key target organ localization and PPI network analysis of key targets. (a) Key target organ localization (Green represents the gene, red represents the organ, and a connecting line indicates that this gene is highly expressed in the organ.) (b) PPI network analysis of key targets (16 nodes and 42 edges. The size of a node is directly proportional to its degree).

**Figure:**
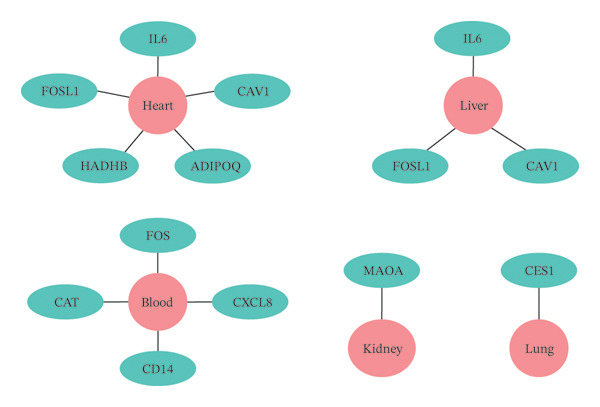
(a)

**Figure:**
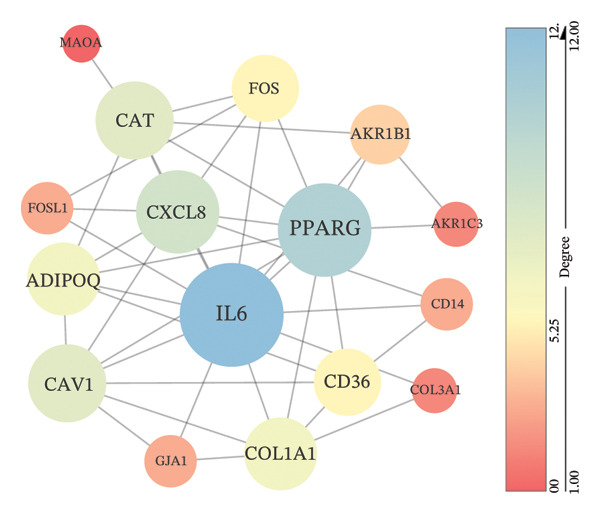
(b)

### 3.7. Construction of PPI Network and Screening of Hub Genes

The PPI network of 18 key targets contained 16 nodes (excluding 2 discrete proteins) and 42 edges (STRING confidence score ≥ 0.4) (Figure 4(b)). The ranking of nodes according to three topological parameters is shown in Table 1. Based on degree ranking, the top five genes in the PPI network were selected as hub genes: *IL-6, PPARγ, CXCL8*, *CAT*, and *CAV1*. These hub genes play a critical role in the therapeutic effects of XHPTS on TED (Figure 4(b)).

**TABLE 1:** The binding energy of hub genes and key active compounds.

Hub genes	PDB ID	Key active compounds	Binding energy (kcal/mol)
PPARγ	1FM9	Quercetin	−7.86
		Luteolin	−8.71

IL‐6	1ALU	Luteolin	−7.93
		Paeoniflorin	−14.87

CXCL8	4XDX	Quercetin	−8.74
		Ellagic acid	−10.16

CAT	1DGF	Naringenin	−8.72
		(+)‐Catechin	−7.6

CAV1	7SC0	Quercetin	−8.53

### 3.8. Immune Microenvironment Analysis

The proportions of 22 types of immune cells were calculated for 27 TED samples and 22 normal samples. After excluding samples with *p* > 0.05, 26 TED samples and 22 normal samples remained. As activated CD4 memory T cells and eosinophils were not detected, a heatmap (Figure 5(a)) and violin plot (Figure 5(b)) were plotted for the remaining 20 immune cells. The analysis identified eight types of immune cells with significant differences (*p* < 0.05) between TED and normal samples: plasma cells, CD4 naïve T cells, monocytes, macrophage M0, macrophage M2, mast cell resting, mast cell activated, and neutrophils. Pearson’s correlation analysis indicated that plasma cells and activated mast cells exhibited the strongest positive correlation (*r* = 0.53), whereas activated mast cells and resting mast cells displayed the strongest negative correlation (*r* = −0.7) (Figure 5(c)). The analysis of the relationships between differential immune cells and hub genes revealed that M0 macrophages and activated mast cells were positively correlated with *IL-6* and *CXCL8* and negatively correlated with *CAV1* and *CAT*. M2 macrophages and resting mast cells were positively correlated with *CAV1*, *CAT*, and *PPARG*, and negatively correlated with *IL-6* and *CXCL8*. Neutrophils showed a positive correlation with *CXCL8* and a negative correlation with *PPARG*. Plasma cells were positively correlated with *IL-6* and negatively correlated with *CAV1* (Figure 5(d)).

FIGURE 5Immune cell analysis. (a) Scoring heatmap of 20 types of immune cells. (b) Violin plot of 20 immune cell infiltration abundances. (c) Correlation analysis of differential immune cells (The redder the circle, the higher the positive correlation; the bluer the circle, the stronger the negative correlation.) (d) Correlation analysis between differential immune cells and hub genes (darker colors in the upper left indicate a more significant correlation; red in the lower right signifies a stronger positive correlation, while blue indicates a stronger negative correlation; ^∗^ *p* < 0.05; ^∗∗^ *p* < 0.01).

**Figure:**
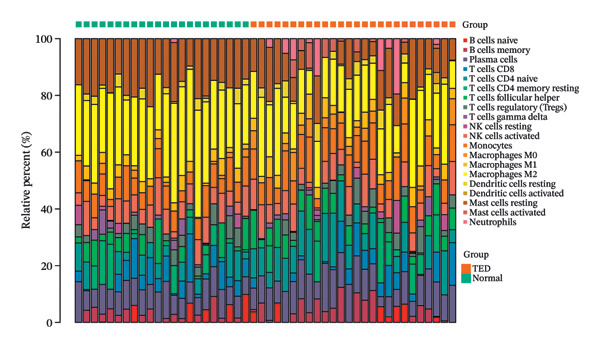
(a)

**Figure:**
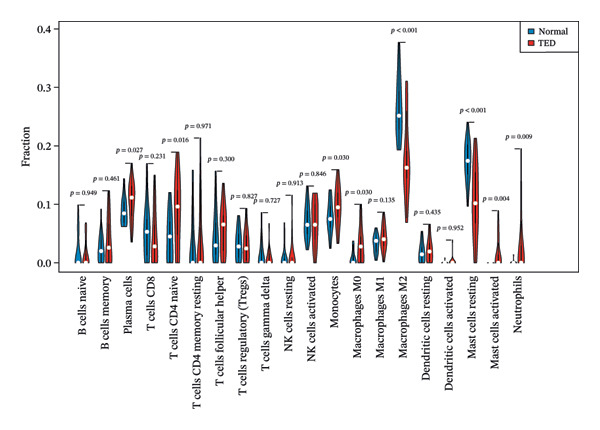
(b)

**Figure:**
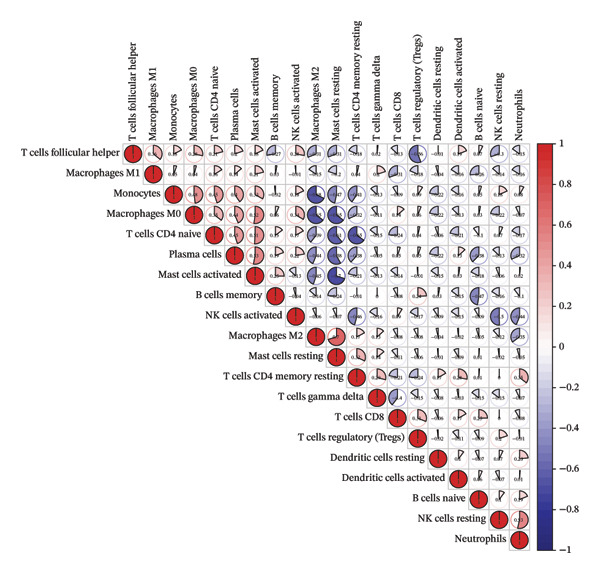
(c)

**Figure:**
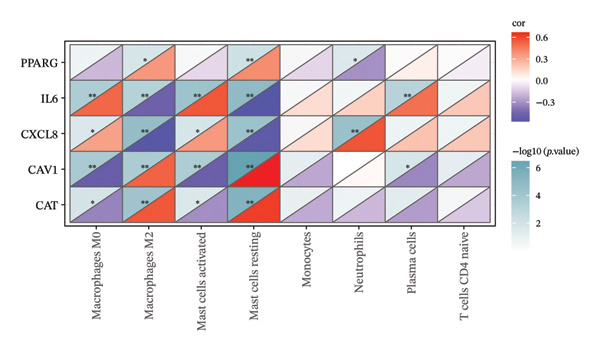
(d)

### 3.9. Analysis of ceRNA Regulatory Network

Five hub genes were predicted to target 970 miRNAs in the miRWalk database and 141 miRNAs in the miRDB database. The intersection of the results from both databases identified 33 shared miRNAs (Figure 6(a)).

FIGURE 6RNA analysis. (a) Venn diagram of miRNA predictions from the miRWalk and miRDB databases. (b) Venn diagram of lncRNA predictions from the Starbase and miRNet databases. (c) mRNA‐miRNA‐lncRNA regulatory network (165 nodes and 321 edges; red circles represent 4 mRNAs, purple squares represent 8 miRNAs, and green diamonds represent 153 lncRNAs).

**Figure:**
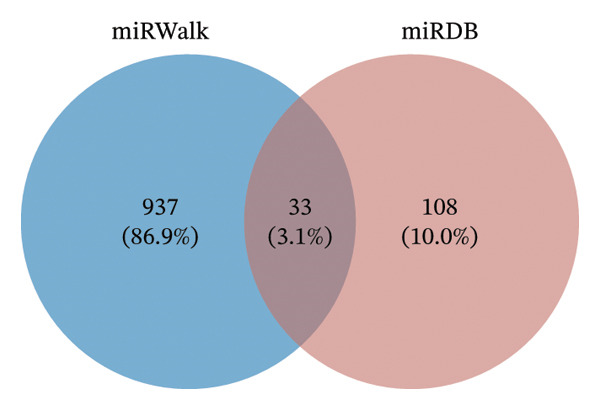
(a)

**Figure:**
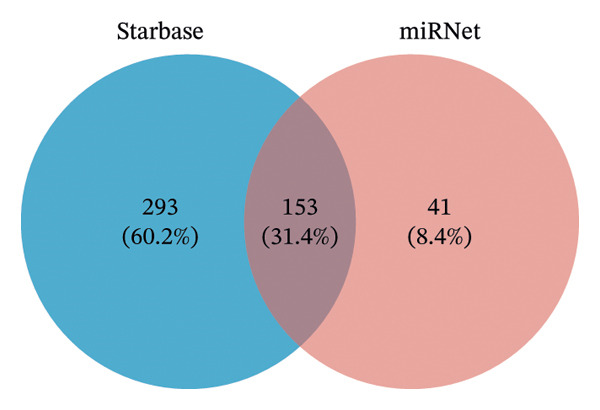
(b)

**Figure:**
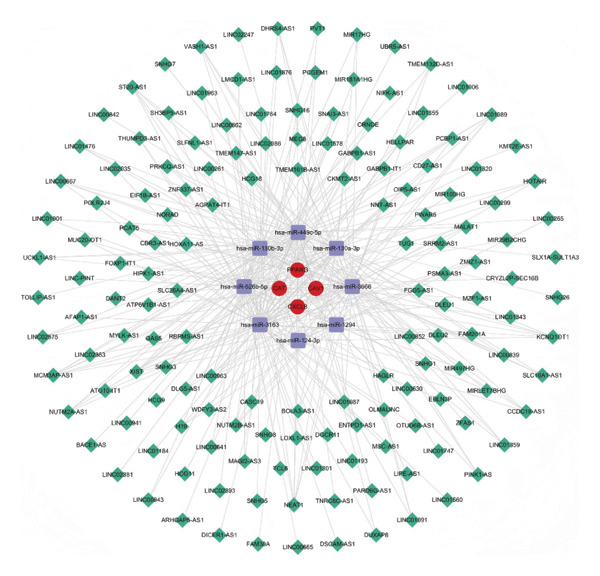
(c)

In the Starbase database, 6 of the 33 shared miRNAs were predicted to interact with 446 lncRNAs, whereas in the miRNet database, 8 shared miRNAs were predicted to interact with 194 lncRNAs. The intersection of these predictions yielded 153 common lncRNAs (Figure 6(b)).

Finally, 8 miRNAs (hsa‐miR‐124‐3p, hsa‐miR‐449c‐5p, hsa‐miR‐130b‐3p, hsa‐miR‐130a‐3p, hsa‐miR‐1294, hsa‐miR‐3666, hsa‐miR‐526b‐5p, and hsa‐miR‐3163) and 4 hub genes (*PPARγ, CAV1, CAT*, and *CXCL8)* were extracted, showing regulatory relationships with 153 lncRNAs. The top 5 lncRNAs were MCM3AP‐AS1, LINC00963, NEAT1, KCNQ1OT1, and XIST. The mRNA‐miRNA‐lncRNA regulatory network is visualized in Figure 6(c).

### 3.10. Molecular Docking

Molecular docking was performed between the 5 hub genes (*IL-6, PPARγ, CXCL8*, *CAT*, and *CAV1*) and the key active ingredients. Lower binding energy indicates a stronger interaction and a more stable molecular conformation. Dimethyl sulfoxide (DMSO) served as a negative control, with binding energy values > −3 kcal/mol. All key active ingredients exhibited minimum binding energies below −7 kcal/mol, demonstrating good docking between the ligand molecules and receptor proteins, indicating favorable binding and strong affinity between the ligand molecules and the receptor proteins. Detailed docking results are summarized in Table 1, and the docking visualizations are presented in Figures 7(a), 7(b), 7(c), 7(d), 7(e), 7(f), 7(g), 7(h), 7(i).

FIGURE 7Molecular docking. (a) PPARγ and quercetin. (b) PPARγ and luteolin. (c) IL‐6 and paeoniflorin. (d) IL‐6 and luteolin. (e) CXCL8 and quercetin. (f) CXCL8 and ellagic acid. (g) CAT and naringenin. (h) CAT and (+)‐catechin. (i) CAV1 and quercetin (the purple ring model represents the active molecular structure; the stick structures near the active molecules are amino acid residues interacting through hydrogen bonding; the yellow dashed lines represent hydrogen bonds formed between the active molecules and amino acid residues).

**Figure:**
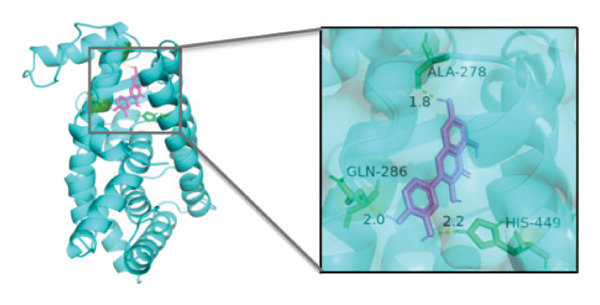
(a)

**Figure:**
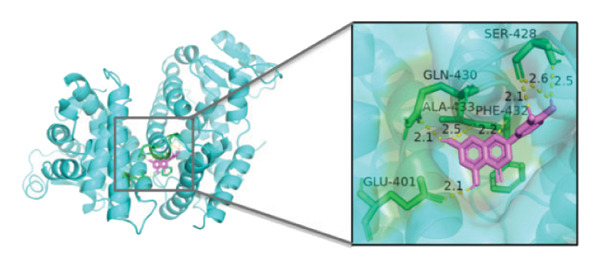
(b)

**Figure:**
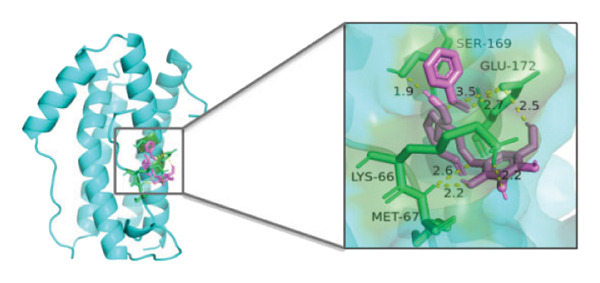
(c)

**Figure:**
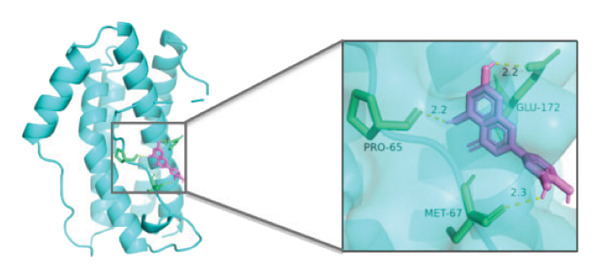
(d)

**Figure:**
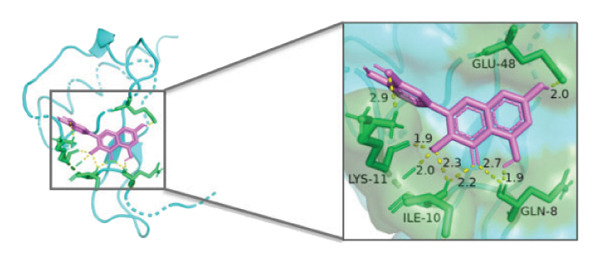
(e)

**Figure:**
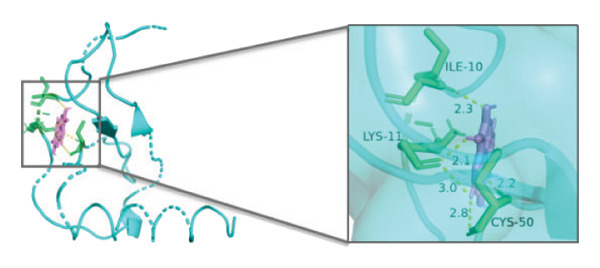
(f)

**Figure:**
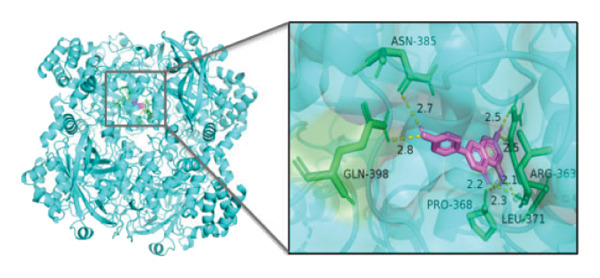
(g)

**Figure:**
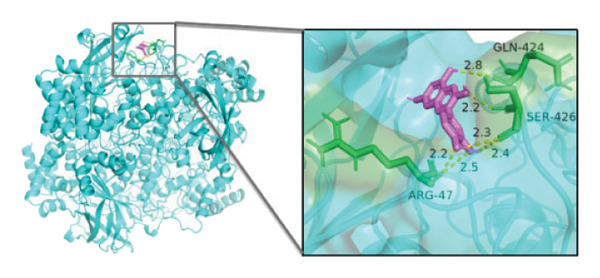
(h)

**Figure:**
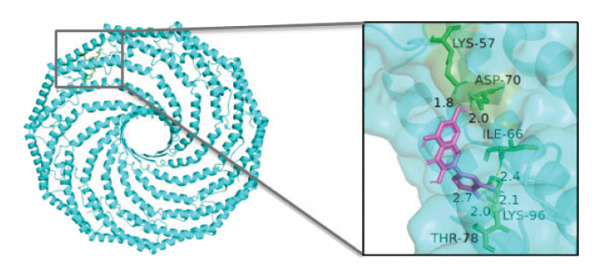
(i)

### 3.11. Molecular Dynamics Simulation

The RMSD results of the protein–ligand complex simulations showed that the protein and ligand reached equilibrium after 20 ns, confirming the stability and reliability of the molecular dynamics simulations (Figure 8(a)). The SASA and Rg curves showed no substantial fluctuations throughout the simulation period, suggesting that the overall compactness and surface exposure of the protein structures in the complexes remained stable (Figures 8(b), 8(c)). The RMSF results showed significant fluctuations in the PPARγ–luteolin complex group, suggesting greater flexibility in the protein structure in this group (Figure 8(d)). Hydrogen bond analysis revealed average hydrogen bond distributions for CXCL8–ellagic acid (1.07), CAT–naringenin (2.00), IL‐6–paeoniflorin (0.89), PPARγ–luteolin (1.57), and CXCL8–quercetin (0.96), indicating the presence of hydrogen bond interactions between the proteins and small molecules (Figure 8(e)). The MMPBSA results demonstrated that the binding free energies of CXCL8–ellagic acid, CAT–naringenin, IL‐6–paeoniflorin, PPARγ–luteolin, and CXCL8–quercetin were negative, with values of −51.923, −102.406, −184.546, −88.880, and −52.400 kJ/mol, respectively. These results indicate stable binding between the proteins and small molecules, with the primary interaction energy being MM (Coulomb electrostatics and van der Waals forces) (Table 2).

FIGURE 8Molecular dynamics simulation. (a) RMSD. (b) SASA. (c) Rg. (d) RMSF. (e) Hydrogen bond analysis.

**Figure:**
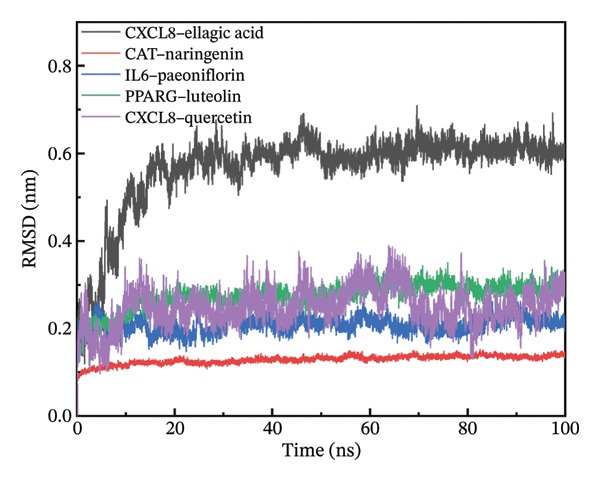
(a)

**Figure:**
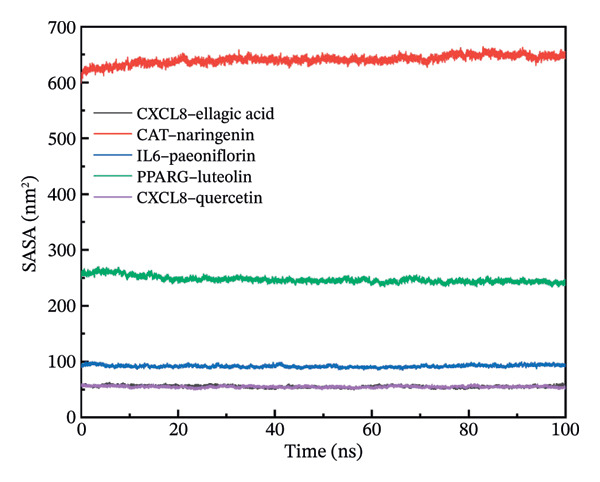
(b)

**Figure:**
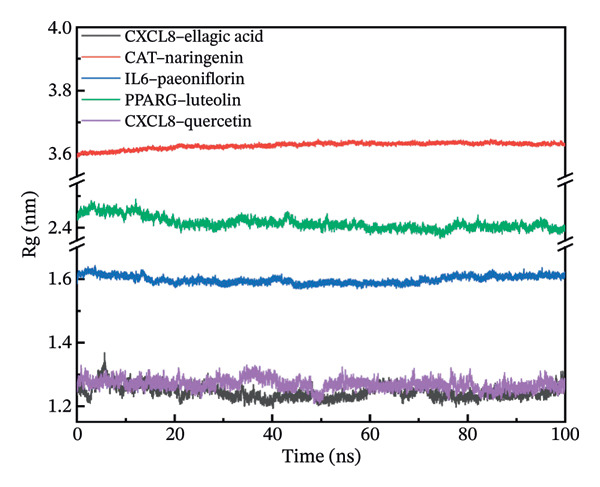
(c)

**Figure:**
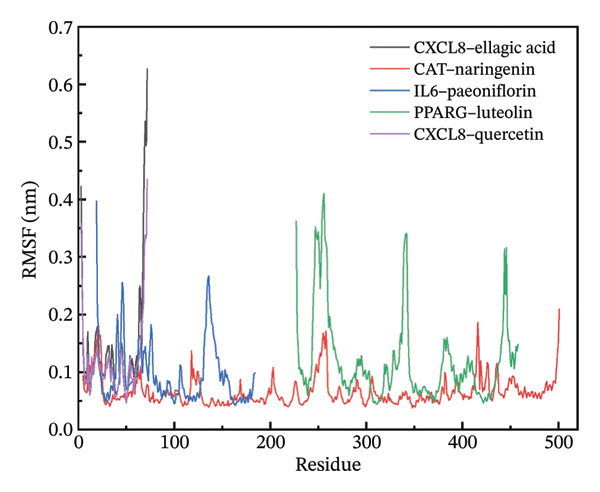
(d)

**Figure:**
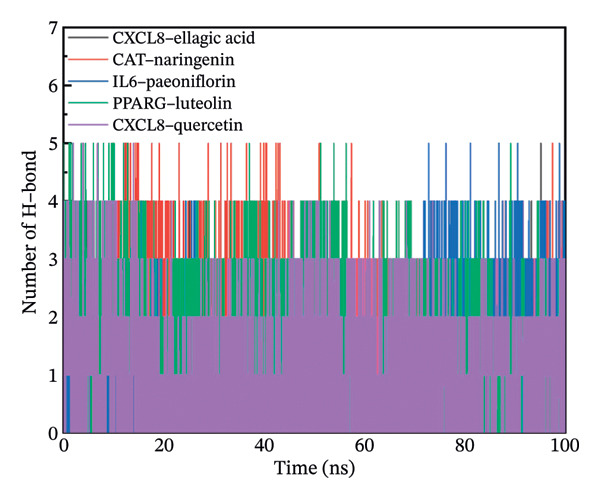
(e)

**TABLE 2:** Analysis of protein ligand MM/PBSA.

Energy(kJ/mol)	CXCL8–ellagic acid	CAT–naringenin	IL‐6–paeoniflorin	PPARγ–luteolin	CXCL8–quercetin
Van der Waals energy	−86.225	−96.143	−92.081	−85.580	−85.942
Electrostatic energy	−11.643	−21.390	−96.877	−57.863	−91.285
Polar solvation energy	45.272	74.663	126.656	127.734	120.910
Nonpolar solvation energy	−12.529	−17.035	−12.488	−16.913	−13.177
Total binding energy	−65.125	−85.972	−94.551	−97.035	−96.257
TΔS	13.202	19.275	20.004	8.156	2.345
Total binding free energy	−51.923	−98.221	−82.261	−88.880	−52.400

## 4. Discussion

TED is a rare autoimmune orbital disease that can seriously affect the quality of life of patients [4]. However, current clinical treatments often provide limited efficacy, highlighting the need to clarify the mechanisms of therapeutic agents and identify more effective interventions. XHPTS has been used in the Endocrinology Department of the First Affiliated Hospital of Henan University of Chinese Medicine as a traditional formula for treating TED with excessive liver fire syndrome. The prescription includes LDC, ZZ, HQ, XKC, JH, QXZ, HYZ, MDP, CS, QD, and BJL. Among these herbs, LDC is characterized by strong bitter and cold properties and is traditionally used to clear excessive heat from the liver and gallbladder. ZZ also exhibits bitter and cold properties and helps dispel heat from the San Jiao, alleviating ocular redness, swelling, and pain caused by rising liver and gallbladder fire. HQ clears heat and dampness, reduces pathogenic fire, and provides detoxifying effects. Together, LDC, ZZ, and HQ constitute the sovereign medicines of the formula, functioning primarily to purge liver fire. XKC acts on the liver and gallbladder meridians and assists in clearing liver fire to improve vision. JH, associated with the liver and lung meridians, calms the liver, enhances visual acuity, and clears heat and toxins. QXZ enters the liver meridian and is used to dissolve ocular turbidity and improve eyesight. These three herbs serve as minister medicines, working synergistically with the sovereign herbs to clear liver heat and restore vision. HYZ detoxifies, resolves phlegm, disperses nodules, and reduces swelling. MDP clears heat, cools the blood, promotes circulation, and disperses stasis. CS reduces liver fire, activates blood circulation, and alleviates pain. QD cools the blood and removes pathogenic spots, while BJL soothes the liver, relieves stagnation, promotes circulation, and dispels wind. These herbs act as adjuvant medicines to address accompanying pathological factors, such as phlegm accumulation and blood stasis. Collectively, the ingredients of XHPTS work to clear liver heat and harmonize the internal environment. Previous studies conducted by our research team have shown that XHPTS is both safe and effective in treating TED, significantly reducing TCM symptom scores, CAS, and NOSPECS grading [9]. However, the active ingredients and therapeutic mechanisms of XHPTS in TED remain unclear. Therefore, this study explores the potential targets and mechanisms of XHPTS in treating TED using NP, molecular docking, and molecular dynamics simulations.

Through database mining and literature review, we identified 248 active ingredient targets for XHPTS and 9758 TED‐related disease targets. Additionally, 659 DEGs were obtained from TED patient samples and normal controls. Among the top upregulated genes were *FOS, EGR1, CHAD, CYR61*, and *CSRNP1*, while the top downregulated genes included *PLIN1, ADIPOQ, LGMN, CIDEC*, and *FABP4*. Both *FOS* and *EGR1* are involved in thyroid hormone production and thyroid development [34, 35]. Research has shown that c‐Fos mRNA levels are elevated in the EOMs of TED patients. Its expression is associated with local pro‐inflammatory responses in TED and influences the response to glucocorticoid treatment [36]. The TGF‐β1/EGR‐1/NOX4 axis is significant in fibrosis development, such as in keloids [37]. CYR61 is an important biomarker linked to TED activity, inducing OFs to produce IL‐6 and CCL20, contributing to orbital inflammation and adipogenesis in TED [38]. Elevated CYR61 expression is more common during the active phase of TED than in the remission phase. Simvastatin has been shown to inhibit CYR61 expression [39]. Downregulated genes, including *PLIN1, ADIPOQ, CIDEC*, and *FABP*4, are involved in lipid metabolism. PLIN1 is a key lipid droplet surface protein, and its downregulation can reduce lipid accumulation and HA production in TED OFs [40]. Basic fibroblast growth factor (BFGF) can significantly stimulate fat production in TED OFs, leading to increased expression of ADIPOQ and CIDEC [41]. Prostaglandin F2*α* (PGF2*α*) and artemisinins can interfere with the lipid accumulation of TED OFs by regulating FABP4, PLIN1, and FABP4 expression [42, 43].

We identified 18 key targets by intersecting 248 active ingredient targets, 9758 disease targets, and 659 DEGs. Through the “drug‐active ingredient‐key target” network, the key active ingredients of XHPTS for treating TED were determined. The highest‐ranked key active ingredients included quercetin, luteolin, paeoniflorin, ellagic acid, naringenin, and (+)‐catechin. Quercetin has been shown to inhibit several key pathological processes in TED, such as inflammation, adipose tissue expansion, OS, and abnormal extracellular matrix (ECM) accumulation [44]. Studies have demonstrated that quercetin significantly reduces the production of IL‐1, IL‐6, IL‐8, TNF‐α, COX‐2, and ICAM‐1 in primary cultured OFs from TED patients [45]. Additionally, quercetin inhibits lipid droplet accumulation by suppressing the upregulation of ROS and heme oxygenase‐1 (HO‐1) in TED OFs and preadipocytes [46]. Moreover, quercetin inhibits fibroblast differentiation induced by cigarette smoke extract and hydrogen peroxide (H_2_O_2_), suppressing OF proliferation and HA release [47]. Catechins can inhibit IL‐8 expression and exert anti‐inflammatory and antioxidant effects by blocking the NF ‐ κ B, p38, and ERK signaling pathways in TED OFs [48]. It can also exert therapeutic potential for TED by regulating the balance of Th17/Treg cells [49]. The synergistic effects of quercetin and catechin may further enhance antioxidant and anti‐inflammatory actions, potentially enhancing therapeutic outcomes in TED [50]. Luteolin inhibits the release of inflammatory cytokines and chemokines from corneal stromal fibroblasts and suppresses the degradation of collagen‐I by blocking signaling pathways, such as TAK‐1, MAPK, c‐Jun, and NF‐κB [51]. Paeoniflorin and naringenin can regulate immune cell functions, such as Th17 and Treg by affecting the expression of signaling pathways, such as PI3K/mTOR and TGF‐β/Smad, providing therapeutic benefits for TED [52, 53]. Ellagic acid downregulates PPARγ expression in a dose‐dependent manner at both mRNA and protein levels, reducing the activity level of TED [54].

GO and KEGG analyses identified the biological functions and signaling pathways associated with the therapeutic effects of the key targets. Construction of the item or pathway–key target interaction network further revealed shared targets across enriched biological processes and signaling pathways, as presented in Section 3.5. The pathological features of TED result from the complex interplay between OS and autoimmune activation [55]. Oxygen free radicals contribute to the proliferation of TED OFs and the production of glycosaminoglycan (GAG) in TED [56]. Elevated levels of ROS and antioxidant enzymes can be observed in OFs, tears, blood, and urine of TED patients. Numerous in vitro and in vivo studies have confirmed the therapeutic effects of antioxidants on TED [57]. The abnormal metabolism of FA in the human body is involved in the pathogenesis of TED. Research has demonstrated differences in FA metabolic characteristics (such as FA mobilization, transport, and digestion) and related pathways (PPARγ and IGF) between TED patients and healthy individuals [58]. There is limited research on the response of TED to alcohol. However, some studies have shown that a small number of thyroid nodule patients without risk factors, such as abnormal thyroid antibodies or smoking, may develop TED‐related complications after receiving percutaneous ethanol injection (PEI) [59]. Additionally, *Klebsiella pneumoniae* has been identified as a potential pathogenic bacterium in TED; its abundance correlates positively with disease severity and may influence TED development by altering FA metabolism and alcohol degradation pathways [60]. Fibrous collagen trimer, collagen fiber, and collagen trimer are all associated with collagen, which serves as an important indicator of the degree of fibrosis in TED. Cyclic peptide 19, derived from the thyrotropin receptor (TSHR), can significantly reduce collagen content in the orbital tissues of TED mice [61]. PDGF expression increases in the orbital tissue of TED [62], stimulating OF proliferation and function and enhancing interactions between TSHR and cytokines/chemokines, which trigger immunopathological responses [63]. Studies have found that the PDGF receptor tyrosine kinase inhibitor imatinib mesylate and PDGF‐BB neutralizing antibody can suppress the inflammatory response in TED orbital tissue [64]. Most TED patients are accompanied by hyperthyroidism, and studies have shown that patients with hyperthyroidism exhibit elevated carotid intima‐media thickness (CIMT), a key marker of atherosclerosis, which decreases as thyroid function improves [65]. While research on the relationship between NAFLD and TED is limited, both conditions share a common pathogenesis involving insulin‐like growth factor‐1 (IGF‐1). The interaction between IGF‐I and TSHR plays a critical role in TED [66]. Meanwhile, IGF‐1 is a key factor in maintaining the pathological homeostasis of NAFLD [67]. Pertussis toxin can inhibit the synergistic phosphorylation of ERK1 and ERK2 in TED fibroblasts and human thyroid cells, blocking the synergistic effect of TSH and IGF‐1 [68]. IL‐17 can enhance the pro‐inflammatory function and ECM production of TED OFs by activating MAPK [69], and it synergistically boosts RANTES expression in OFs when combined with CD40L [70]. Studies have shown that IL‐17 levels correlate with CAS and visual acuity in TED patients [71]. The monoclonal antibody teprotumumab, which targets IGF‐1R, has therapeutic effects on TED partly by regulating IL‐17A expression levels [72].

In terms of shared targets involved in the aforementioned biological functions and signaling pathways, ADIPOQ is associated with adipogenesis and differentiation in orbital tissue [41]. BFGF can significantly stimulate fat production in TED OFs, leading to increased expression of ADIPOQ and CIDEC [73]. Catalase (CAT) is involved in ethanol metabolism [74] and the breakdown of ROS [75] in the human body. Studies have shown that CAT activity is elevated in the blood of TED patients [76]. The increased concentration of alcohol and ROS can activate c‐FOS phosphorylation [77, 78], and elevated c‐Fos mRNA levels are found in TED EOM. The expression of c‐Fos is associated with the local pro‐inflammatory response of TED and participates in the patient’s response to glucocorticoids [36]. COL1A1 and COL3A1 are fibrosis markers [79]. Disulfiram inhibits fibrosis in TED by reducing COL1A1 expression at the mRNA and protein levels [80]. CD14, mainly expressed in macrophages and neutrophils, shows higher methylation levels in TED [81]. CD14^+^CD16^+^monocytes are involved in TED pathogenesis by secreting higher levels of B‐cell activating factor [82]. Research has shown that CXCL8 expression is elevated in almost all cell types within the orbital connective tissue (OCT) of TED patients, with enhanced interactions between OFs and CXCL8 [83]. IL‐6 can increase the expression of TSH‐R on TED OFs. Some redox signaling pathways can exacerbate the inflammation of TED by upregulating IL‐6 and promoting adipogenesis and fibrosis of orbital tissue [84]. Elevated IL‐6 levels have been detected in both peripheral blood and OCT of patients with TED [85], and these levels show a positive correlation with the CAS in active TED [86]. Tocilizumab, an IL‐6 receptor inhibitor, has demonstrated safety and efficacy in refractory TED, significantly reducing CAS, thyroid‐stimulating immunoglobulin (TSI), Hertel scores, eye protrusion, and shortening the duration of diplopia [85, 87]. PPARγ, a key regulator of lipid metabolism and inflammation, is expressed in EOM cells, OFs, preorbital adipocytes, and thyroid cells in TED patients. It promotes OF differentiation into adipocytes [88] and induces the production of adiponectin (APN) [89]. Additionally, PPARγ regulates the production of recombinant hyaluronan synthase (HAS) in TED [90].

Organ localization analysis of 18 key targets revealed that IL‐6, FOSL1, and CAV1 are highly expressed in the liver. The roles of IL‐6 and FOSL1 in TED have already been discussed. CAV1 plays a critical role in OS in TED adipocytes. The OCT in TED shows increased hypoxia inducible factor‐1 (HIF‐1) in a state of redox imbalance, and HIF‐1 directly regulates the transcription of CAV1, contributing to tissue inflammation and edema [91]. TCM suggests that “the eyes are the window of the liver,” highlighting the close relationship between the liver and the eyes. Abnormal liver function has been clinically associated with the onset of TED [92], and therapeutic approaches based on liver regulation have shown favorable outcomes in managing TED [93]. Modern research supports this, showing that the expression pattern of functional TSHR genes in liver tissue mirrors that in the thyroid [94]. Liver dysfunction influences the occurrence and progression of TED through mechanisms involving metabolism, immune regulation, and other factors [95, 96]. The liver can secrete hepatocyte growth factor (HGF) and fibroblast growth factor 21 (FGF‐21). Nowak et al. reported significantly increased HGF levels in active TED compared with healthy controls, with reductions observed after glucocorticoid treatment [97]. Ueland et al. found elevated FGF‐21 levels in TED patients compared to those without eye signs and healthy individuals, suggesting that FGF‐21 may serve as a biomarker for TED diagnosis and treatment [98]. Clinically, some TED patients exhibit abnormal liver function indicators. High free thyroxine (FT4) and thyroid‐stimulating hormone receptor antibody (TRAb) levels, low TSH levels, and advanced age are independent risk factors for liver injury in TED patients [99]. These findings suggest a significant connection between liver dysfunction and TED. This study also found that IL‐6, FOSL1, CAV1, and ADIPOQ are highly expressed in the heart; MAOA is highly expressed in the kidneys; CES1 is highly expressed in the lungs; and CAT, CD14, CXCL8, and FOS are highly expressed in the blood. The heart regulates blood vessels and controls the circulation of blood throughout the body. When blood circulation is smooth and mental function is clear, the eyes remain healthy, and vision is sharp. Disrupting heart function and blood circulation can contribute to TED progression [100]. Clinically, treating TED from the perspective of the “heart” has shown positive outcomes [101]. In the advanced stages of TED, the disease often affects the kidneys. Professor Wang Xu advocates nourishing both the liver and kidneys to treat TED [102]. Classical TCM literature such as *Lingshu: Jue Qi* states, “When qi is deficient, vision becomes unclear.” The lungs, regarded as the source of qi, are fundamental for maintaining vision clarity. If lung qi is obstructed, the distribution of qi, blood, and body fluids to the eyes becomes impaired, potentially causing blurred vision and eyelid swelling. Based on this theoretical framework, some scholars have proposed treating TED from the perspective of lung regulation [103]. However, there is limited research in modern medicine directly linking the heart, kidneys, lungs, and TED, and further studies are necessary to explore these connections.

Through PPI analysis of key targets, five hub genes were identified as critical in the treatment of TED with XHPTS: *IL-6, PPARγ, CXCL8, CAT*, and *CAV1*. The roles of these hub genes in TED have been discussed in previous sections.

Immune microenvironment analysis revealed significant differences in immune cell profiles between TED and healthy samples, along with notable correlations both among differential immune cells and between these cells and the hub genes, as presented in Section 3.8. Research indicates that plasma cells contribute to TED by producing TRAb [53], while rituximab can exert therapeutic effects on TED by consuming plasma cells [104]. Inflammatory cell infiltration, primarily composed of activated T cells, has been observed in the orbital tissues of TED patients. Compared to healthy individuals, TED patients exhibit more naïve active T cells [105]. Monocytes can also participate in the pathogenesis of TED through various pathways. TED patients have a higher positive rate of TSHR in monocytes compared to other cells [106], and monocyte levels are positively correlated with TED CAS [53]. Macrophages influence TED immune metabolism through their distinct phenotypes. M1 macrophages release pro‐inflammatory cytokines, such as TNF, IL‐1, and IL‐6, while M2 macrophages secrete anti‐inflammatory cytokines, such as IL‐10 and TGF‐β, which are involved in the development of TED [107]. Research has shown that compared to healthy individuals, TED patients exhibit an increase in M1 macrophages and a decrease in M2 macrophages in preorbital tissue. CD86^+^ M1 macrophages are predominant in active TED patients, while stable TED patients have more CD163^+^ M2 macrophages. Additionally, an increase in M0 macrophages has been observed in the orbital tissues of TED patients compared to healthy individuals [108]. Mast cells can activate OFs and enhance their chemotaxis by regulating orbital tissue inflammation response, OS response, and various signaling pathways, promoting tissue remodeling processes, such as fat generation and fibrosis in OCT and EOM [88, 108–110]. Mast cell numbers rise near connective tissue, OFs, and adipocytes in the orbital muscles of TED patients [109]. Therapeutic agents, such as dexamethasone and anti‐IL‐4 receptor antibodies, have been shown to block mast cell functions, providing therapeutic benefits for TED [111]. The activation and release of neutrophil extracellular traps can induce the formation of autoantibodies. In vitro studies have shown that extracellular S100A11 demethylation enhances TED inflammatory response by inducing neutrophil pro‐inflammatory cytokines [112, 113]. Clinical studies have shown that the neutrophil‐to‐lymphocyte ratio (NLR) of TED patients is higher than that of healthy individuals, and the NLR of active TED patients exceeds that of inactive patients [114]. The CAS [115], MR imaging parameters [114], and thyroid function [116] of TED are all related to NLR. The interactions between various immune cells also influence TED progression. For instance, the interaction between mast cells, monocytes, and macrophages in TED increases the secretion of PDGF‐AB and PDGF‐BB, which in turn activates OFs. The transition of monocytes into macrophages in TED orbital tissue significantly affects inflammation, adipogenesis, and fibrosis, making it crucial for evaluating disease stage, progression, and prognosis [110, 117]. As identified earlier, the key active ingredients of XHPTS—quercetin, luteolin, paeoniflorin, ellagic acid, naringenin, and (+)‐catechin—also exhibit regulatory effects on relevant immune cells and target genes. For instance, NRICM102, which contains quercetin, has been shown to reduce the activation of neutrophils and monocytes, along with the expression of cytokines, such as TNF‐α, IL‐1β, IL‐6, and IL‐8 [118]. Quercetin promotes M2 macrophage polarization via modulation of the PI3K/Akt/NF‐κB signaling pathway [119]. Naringenin influences T cell–mediated autoimmune diseases through the regulation of genes including CAT [120]. Luteolin can inhibit skin infiltration of macrophages, T cells, and neutrophils. The expression of cytokines is downregulated, such as IL‐6, IL‐1β, TNF‐α, IL‐17A, and IL‐23, in mouse skin and ocular blood [121].

Analysis of the ceRNA regulatory network revealed that 153 lncRNAs interact with eight miRNAs and four hub genes. Epigenetics, particularly the roles of miRNAs and lncRNAs, plays a crucial part in the pathogenesis of TED. These noncoding RNAs regulate immune cell function, inflammatory response, lipid accumulation, OS, and other processes, offering potential diagnostic value for TED and related diseases [122]. MiR‐130b‐3p influences TED by modulating the inflammatory response, regulating PPARγ expression and the IGF‐1/IGF‐1R signaling pathway [123, 124]. MiR‐130a reduces lipid accumulation in TED by activating AMPK, inhibiting adipogenic gene activity, and suppressing FA synthesis [125]. MiR‐1294 contributes to TED pathogenesis by regulating IGF1R expression [126]. Cell ferroptosis has been linked to TED progression [127]. Research has demonstrated differential expression of ferroptosis‐related RNAs in OFs between TED patients and healthy individuals [128]. MiR‐124‐3p inhibits ferroptosis by downregulating SteaP3 expression [129], while miR‐130b‐3p modulates ferroptosis by regulating the AMPK/mTOR signaling pathway and directly targeting ACSL4 [130]. Although studies directly linking lncRNAs to TED are limited, several lncRNAs identified in our ceRNA network have established roles in pathological processes relevant to TED. MCM3AP‐AS1 attenuates inflammation, OS, and mitochondrial dysfunction through the miR‐501‐3p/CADM1/STAT3 axis [131]. Linc00963 [132], NEAT1 [133], and Kcnq1ot1 [134] are linked to ferroptosis in cells, fibrosis of organs, and tissue inflammatory responses [135, 136]. XIST can enhance M2 macrophage polarization [137] and promote Th17 cell differentiation [138], both of which participate in the development and progression of TED.

The molecular docking results between the hub genes and key active ingredients in this study indicated that quercetin, luteolin, paeoniflorin, ellagic acid, naringenin, and (+)‐catechin show strong affinity for IL‐6, PPARγ, CXCL8, CAT, and CAV1. Molecular dynamics simulations further supported these findings. Numerous studies have demonstrated that quercetin significantly inhibits the expression of IL‐6 mRNA and PPARγ protein in orbital tissues of TED patients. Quercetin also modulates CAT [139], CXCL8 [140], and Cav1 [141]. Luteolin binds directly to PPARγ, enhancing its expression and function [142], and inhibiting LPS‐induced inflammation by reducing IL‐6 and CXCL8 production [143]. In addition, luteolin can reduce intracellular ROS levels and increase CAT expression [144]. Paeoniflorin can reduce the mRNA or protein expression of IL‐6 and CXCL8 [145], activate PPARγ expression [146], and inhibit the OS process by regulating CAT levels [147]. Ellagic acid can improve the severity of diseases by affecting the expression of PPARγ, IL‐6, and CAT [148]. Naringenin inhibits IL‐6 [149], while activating PPARγ [150] and CAT [151]. Catechin enhances PPARγ mRNA expression [152], induces uncoupling of eNOS with CAV1 [153], reduces IL‐6 expression and secretion [154], and regulates CAT activity [155]. Thus, the key active ingredients mentioned above may exert therapeutic effects on TED by modulating the expression and function of the hub genes.

As a complex autoimmune disease, the pathogenesis and precise biomarkers of TED still necessitate further exploration. In recent years, multiomics techniques have functioned as powerful tools for novel biomarkers to assist in TED diagnosis, activity assessment, and prognosis judgment [156, 157]. Our study adopted a “dry experiment multi omics” strategy. By integrating comprehensive analysis methods, such as network pharmacology, transcriptomics (GSE58331 DEGs), proteomics (PPI), organ localization, immune microenvironment, and epigenetics (ceRNA), we simulated the “multi‐component, multi‐target, and multi‐pathway” mode of action of XHPTS at the computational level. It is particularly noteworthy that the model was validated through molecular docking and molecular dynamics simulations. This strategy complements the current trend in multiomics research. It is predominantly intended to identify verifiable core targets from massive data at lower cost and with higher efficiency. Specifically, this study contextualizes the key drug components of XHPTS (e.g., quercetin and luteolin) and the hub genes associated with TED (e.g., IL‐6 and PPARγ) within the framework of established biomarkers—including signaling pathways, immune microenvironment, and post‐transcriptional regulatory networks—to elucidate the mechanistic basis of the therapeutic effects of TCM formulations. Meanwhile, GTEx analysis revealed genes that are specifically highly expressed in the liver in TED. This finding may help to bridge the holistic perspective of TCM theory and multiomics insights into TED pathophysiology.

In this study, the drug components we screened and their potential modes of action can also be regarded as a computationally derived priority list of “potential biomarkers and intervention targets for TED.” Specifically, this study predicts that the active components of XHPTS (e.g., quercetin and luteolin) can synergistically act on core targets (e.g., IL‐6, PPARγ, CXCL8, CAT, and CAV1), regulate multiple pathways (such as IL‐17 signaling, lipid metabolism, and OS), and influence immune cells along with the ceRNA network. Through these integrated actions, they exert multifaceted effects—including anti‐inflammatory, antioxidant, metabolic regulation, and immunomodulatory activities—thereby contributing to the treatment of TED. To further validate these findings, we plan to employ cellular models to examine the impact of key XHPTS components on hub gene expression and their downstream functions. In parallel, animal models will be utilized to evaluate the ameliorative effects of XHPTS on typical TED pathological alterations and its regulatory influence on the predicted network. Thus, this study provides a solid theoretical foundation and clear directions for these subsequent in‐depth investigations.

**FIGURE 9:**
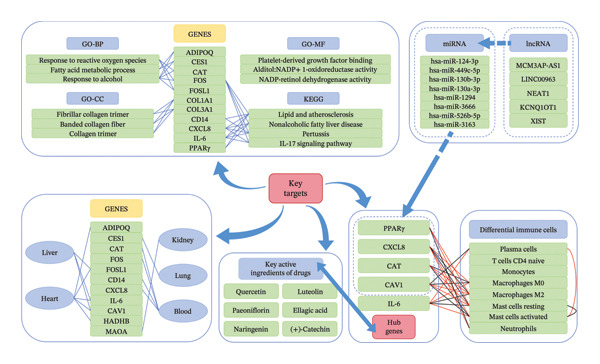
Analysis result pattern diagram. Blue arrow: relevant analysis based on key targets; blue dashed arrow: regulatory relationship; blue double arrows: molecular docking and molecular dynamics simulation. Blue line: correlation; red line: positive correlation; black line: negative correlation.

The pattern diagram of the above analysis results is shown in (Figure 9).

## 5. Conclusion

This study is the first to systematically investigate the active components and potential mechanisms of XHPTS in the treatment of TED using network pharmacology, molecular docking, and molecular dynamics simulations. The key active ingredients of XHPTS (quercetin, luteolin, paeoniflorin, ellagic acid, naringenin, and (+)‐catechin) exert therapeutic effects on TED by regulating the hub genes (IL‐6, PPARγ, CXCL8, CAT, and CAV1). XHPTS acts on TED through various biological processes, such as the response to ROS, FA metabolic processes, and response to alcohol. It also influences cellular components, including fibrous collagen trimer and banded collagen fibers; molecular functions, such as PDGF binding; and signaling pathways, such as lipid and atherosclerosis, NAFLD, pertussis, and IL‐17. Shared targets, including ADIPOQ, CES1, CAT, FOS, FOSL1, COL3A1, CD14, CXCL8, IL‐6, and PPARG, participate in these pathways and contribute to TED pathogenesis. Organ localization analysis further indicated that XHPTS may exert therapeutic effects primarily through liver‐related mechanisms, although associations with the heart, kidneys, and lungs were also observed. Differential gene expression and immune infiltration analyses identified distinct immune cell populations associated with TED and revealed correlations between differential immune cells and hub genes. In addition, lncRNAs and miRNAs identified in the ceRNA network demonstrated regulatory relationships with these hub genes. Molecular docking and dynamics simulations confirmed strong binding affinities between the key active ingredients and the hub genes, providing further support for the predicted interactions. Despite these findings, this study has limitations. The reliability of the results depends on the completeness and accuracy of publicly available databases, and the predicted mechanisms require validation through in vitro, in vivo, and clinical studies.

In conclusion, XHPTS therapy for TED displays multicomponent, multitarget, and multipathway characteristics. These insights provide a useful foundation for further experimental research and offer a scientific basis for the clinical application of XHPTS in the treatment of TED.

## Author Contributions

Shuxun Yan and Ping Wang conceived and designed this study, while Yu Fu and Ying Wang optimized the design. Xin Shang extracted the data. Ping Wang and Ruiyan Liu checked the data and conducted statistical analysis and jointly wrote the manuscript. Shuxun Yan, Yu Fu, and Ying Wang reviewed the manuscript. All authors have provided constructive contributions to this paper.

## Funding

This work was supported by the Youth Science Fund Project of Henan Provincial Natural Science Foundation (252300420619), the Joint Construction of Scientific Research Project by the National Traditional Chinese Medicine Inheritance and Innovation Center of Henan Provincial Health Commission (2024ZXZX1175), the Henan Province Traditional Chinese Medicine Inheritance and Innovation Talent Project (Zhongjing Project), the Top Notch Traditional Chinese Medicine Talents Project (2022ZYBJ01), and the Zhengzhou Collaborative Innovation Special Project (2023XTCX048).

## Ethics Statement

The authors have nothing to report.

## Consent

The authors have nothing to report.

## Conflicts of Interest

The authors declare no conflicts of interest.

## Data Availability

All data generated or analyzed in this study are included in the article and its supporting information files, and may be obtained from the corresponding author upon reasonable request.
